# Epidemiological Profile of Salivary Gland Tumors in Southern Iranian Population: A Retrospective Study of 405 Cases

**DOI:** 10.1155/2023/8844535

**Published:** 2023-11-20

**Authors:** Hamid Ghaderi, Estie Kruger, Simin Ahmadvand, Yousef Mohammadi, Bijan Khademi, Abbas Ghaderi

**Affiliations:** ^1^Faculty of Science, School of Human Science, University of Western Australia, Australia; ^2^West Wimmera Health Service, Nhill, Victoria 3418, Australia; ^3^Shiraz Institute for Cancer Research, Shiraz University of Medical Sciences, Shiraz, Iran; ^4^Department of Otolaryngology, Shiraz University of Medical Sciences, Shiraz, Iran

## Abstract

**Aims:**

Salivary gland tumors (SGTs) are a rare and diverse group of tumors that account for 3 to 10% of all head and neck malignancies. We aimed to conduct a comprehensive epidemiological analysis of SGTs in the south of Iran and compare the findings with previous reports from Iran and other parts of the world.

**Methods:**

Using a retrospective study, 405 patients diagnosed with SGTs were observed over an eight-year period between April 2013 and October 2021 in Shiraz, Iran. Patients' demographic and clinicopathological features were obtained from patients' records. Quantitative and descriptive data analysis was performed using SPSS software.

**Results:**

There were 302 benign (74.5%) and 103 (25.4%) malignant SGTs. Pleomorphic adenoma and Warthin's tumors were the most common benign SGTs (70.5% and 21.5%, respectively). The most common malignant SGTs were mucoepidermoid carcinoma and adenoid cystic carcinoma (26.2% and 22.3%, respectively). There was a statistically significant association between tumor origin and its malignancy status (*p* < 0.001). In addition, the results indicated that benign tumors were most commonly detected in the parotid gland (*p* < 0.05). The benign tumors were more frequently observed among the younger population (*p* = 0.006).

**Conclusion:**

In summary, the findings of the current study were mainly consistent with the previous reports from Iran and the rest of the world. Benign tumors were the most prevalent type of SGTs, and the parotid gland was the most common site. While the majority of cases that developed from the major salivary glands were benign, all the minor SGTs were malignant. Older patients were more likely to develop malignant tumors compared to younger ones. This study provides insights into the prevalence, age-related incidence, gender distribution, and geographic variation of salivary gland tumors. This can be instrumental to develop a guideline for screening, diagnosis, and determining an optimal treatment.

## 1. Introduction

The salivary glands are exocrine glands responsible for the production, modification, and secretion of saliva into the oral cavity. Salivary glands are present throughout the oral and maxillofacial region, including the three paired major salivary glands, as well as 600–1000 minor salivary glands that exist in the oral cavity and aerodigestive tract [1, 2]. Major and minor salivary glands are associated with a diverse group of lesions displaying a wide variety of morphological and biological characteristics [1]. Salivary gland tumors (SGTs) are rare tumors that account for 3 to 10% of all head and neck malignancies [3–5] with an annual incidence estimated from 0.4 to 13.5 cases per 100,000 people worldwide [1, 6, 7].

Owing to tumor rarity and its wide morphological diversity, the etiology of SGTs is poorly understood. Similar to other types of head and neck cancers, SGTs are linked with smoking and alcohol consumption. However, this association is confined to certain tumor locations and a few histological subtypes [8–10]. Additionally, high consumption of processed meat, low-vegetable and high-animal fat diets, obesity, and occupational radiation exposure are possible risk factors for SGTs [11–13]. Epstein-Barr virus, immunodeficiency, HIV infection, and radiotherapy are also associated with an elevated risk of developing SGTs [14–18].

The fourth *Classification of Head and Neck Tumors* lists 33 different histological subtypes of SGT (although in the fifth and the latest edition, the number is more than 33), with pleomorphic adenoma, Warthin tumor, mucoepidermoid carcinoma, and adenoid cystic carcinoma being the most prevalent types [19]. The majority of SGTs are benign and most commonly detected in major salivary glands [20–22].

Despite the fact that several previous studies investigated the frequency and distribution of SGTs in Iran [3, 23, 24] and other parts of the world [6, 7, 20, 21], in the absence of cancer registries, epidemiological data on SGTs are still not well documented. Widespread heterogeneity in the incidence and prevalence of SGTs can also be influenced by geographical and racial variables [25–27].

The aims of the study are to provide a current and comprehensive epidemiological analysis of SGTs in the south of Iran and compare the findings with previous epidemiological data from Iran and other parts of the world.

## 2. Methods

This retrospective study included the epidemiological data analysis of patients diagnosed with primary SGTs, who were referred to the ear, nose, and throat departments of Khalili and Ghadir Mother and Child hospitals (both affiliated with Shiraz University of Medical Sciences) between April 2013 and October 2021. Shiraz is located in the south part of Iran and the capital city of the Fars province and is the referral center for a considerable proportion of patients from the southern provinces of Iran, including Bushehr, Khouzestan, Hormozgan, Kohgiluyeh, and Boyer-Ahmad. Patients' demographic information, such as age, gender, anatomical location, and other pathological data including lymph node involvement, peripheral invasion, stage, and grade of SGTs, was collected from the patients' hospital records or was retrieved from their pathological reports. The study was authorised by the Shiraz University of Medical Sciences' ethical committee (IR.SUMS.REC.1400.869) as well as the University of Western Australia ethics committee (2022/ET000277).

Variables included in the data were patients' gender, age, and their SGT features, which included tumor type (benign or malignant), anatomical location, tumor subtype, and tumor size. The Statistical Package for the Social Sciences (SPSS) software (SPSS version X20 Inc., Chicago, IL, USA) was used to conduct descriptive and quantitative data analysis. For continuous variables, results were reported as mean ± standard deviation or mean and range, and for categorical variables, data were reported as absolute count and percentage values. Person's chi-square test, Student *T*-test, and Fisher's exact test were used to evaluate the association between variables.

### 2.1. Exclusion Criteria

After collecting all pathology reports, the datasheet was checked, and any duplicated cases (especially due to multiple recurrences) with the same information and unclear diagnoses were excluded. Cases with diagnoses other than SGTs (including salivary duct cyst, chronic sialadenitis, salivary polyps, and those with no evidence of malignancy), metastatic carcinoma, recurrent tumors, minor SGTs of the nasopharynx, and nonspecific SGTs were excluded. Overall, 269 cases were removed, leaving 405 patients eligible for the final analysis.

## 3. Results

This study included 405 patients diagnosed with primary tumors originating from major salivary and minor SGs of the oral cavity over 8 years, from two referral hospitals in the city of Shiraz. The majority of cases—54.1% or 219 cases—were involving female SGTs, compared with 186 cases of male SGTs, with a female-to-male ratio of 1.17 : 1 (Table 1).

### 3.1. Tumor Type

This study included 302 (74.5%) cases of benign and 103 (25.4%) cases of malignant SGTs with a benign-to-malignant ratio of 2.9 : 1. A similar distribution pattern was observed in each gender group with 25.8% of males and 25.1% of females having malignant tumors (Table 2). The mean age of patients with malignant tumors was 52.3 years (ranged from 11 to 95 years), which was significantly higher than that of benign tumors (45.7, range 7-83 years), *p* = 0.001.

All of the SGT samples were distributed among 6 benign and 17 malignant histological subtypes (Table 2). The majority of benign tumors (70.5%, *n* = 213) were diagnosed with pleomorphic adenoma. Warthin's tumors were the second most common benign tumor (21.5%, *n* = 65). Monomorphic adenoma (0.6%) was the least frequent type of benign tumor. Mucoepidermoid carcinoma, adenoid cystic carcinoma, and acinic cell carcinoma were the most prevalent malignant tumors, accounting for 26.2%, 22.3%, and 13.5% of malignant tumors, respectively. Among the cases, adenosquamous cell carcinoma, small cell neuroendocrine carcinoma, adenocarcinoma, papillary adenocarcinoma, papillary carcinoma, and oncocytic carcinoma were the less prevalent types of malignant tumors.

The histological distribution of SGTs exhibited a significant gender disparity. For instance, Warthin's tumor, mucoepidermoid carcinoma, and salivary duct carcinoma were more common in males, whereas pleomorphic adenoma, basal cell adenoma, and adenoid cystic carcinoma were more common in females (Table 2).

### 3.2. Tumor Location

**Figure 1:**
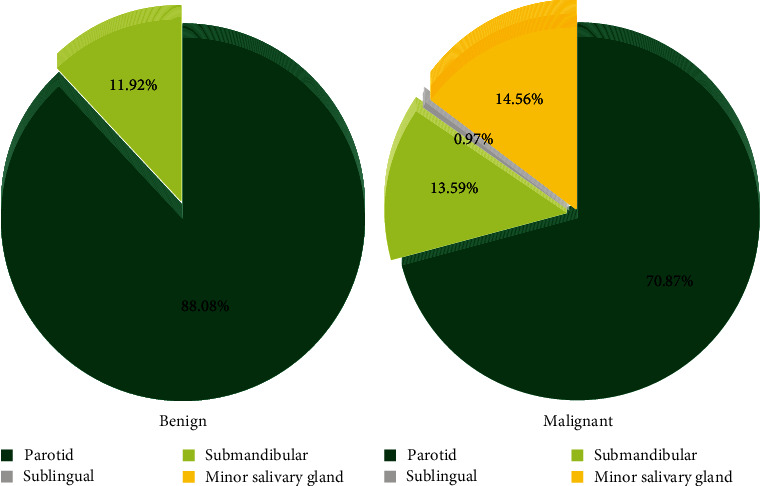
Distribution of (a) benign and (b) malignant SGTs according to the site of involvement. When the tumor's behaviour (malignant vs. benign) was investigated, it was found that benign tumors most commonly developed from the parotid gland.

The study population included 390 (96.2%) major and 15 (3.7%) minor SGTs. Comprising 83.7% of all SGTs, the parotid gland was the most frequently reported tumor site, followed by the submandibular gland with 12.3% of all SGTs (Table 3 and Figure 1). When patients were divided according to their gender, 89.2% and 8.1% of males' SGTs and 79.0% and 16% of females' SGTs occurred in the parotid and submandibular glands, respectively.

**Figure 2:**
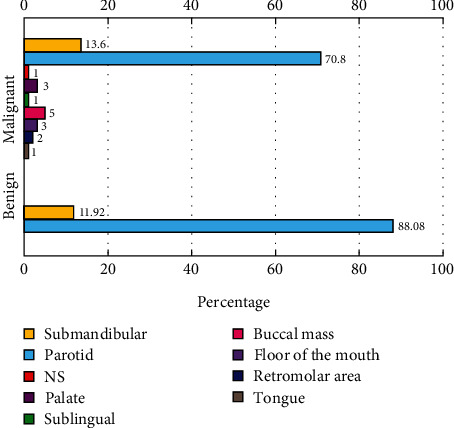
Primary site of involvement of benign and malignant SGTs.

Most of the minor SGTs originated from the buccal mucosa (33.3%), followed by the floor of the mouth (20%), palate (20%), retromolar areas (13.3%), and tongue (6.6%). In addition, our study included one case of malignant SGT in the sublingual gland. There was one case of minor SGT with an unspecified anatomic location (Table 3 and Figure 2).

While the majority of the parotid (78.4%) and submandibular gland (72.0%) tumors were benign, all minor SGTs were malignant. In addition, the only case of sublingual gland tumor was malignant. Overall, there was a statistically significant association between tumor origin and its malignancy status; while the majority of cases originating from the major salivary glands were benign tumors, all cases of minor salivary glands were malignant (*p* < 0.001). In addition, when the tumor's behaviour (malignant vs. benign) was investigated, it was found that benign tumors most commonly developed from the parotid gland (*p* < 0.05) (Table 4).

### 3.3. Age and SGTs

The mean age of patients at the time of diagnosis was 47.4 ± 16.2 years (ranged from 7 to 95 years). The mean age of males and females was 51.1 and 44.2 years, respectively. The distribution of benign and malignant SGTs across different age groups revealed that the majority of cases occurred in people aged 40-60 years, but there were some minor differences. For instance, pleomorphic adenoma was most commonly found in individuals aged 20-40 years, comprising 48.3% of all pleomorphic adenoma cases. The most prevalent age range for Warthin's tumor, the second most common SGT, was 60-80 years (49.2%) (Table 5). Overall, among the different age groups, the parotid gland was the most affected site.

**Figure 3:**
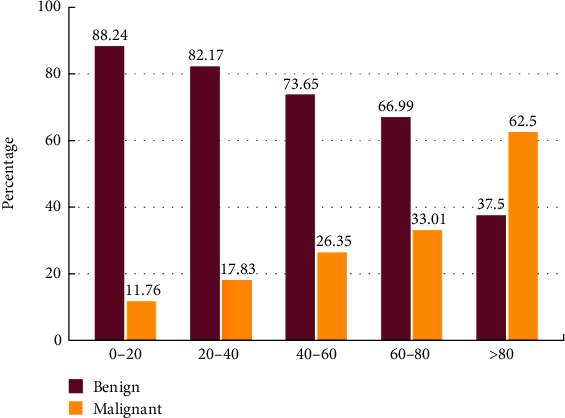
Distribution of benign and malignant SGTs in different age groups (20 year intervals).

In addition, there was a statistically significant association between tumor type (benign vs. malignant) and age of the patient at diagnosis. While the younger population was more prone to benign tumors, a higher percentage of older ones suffered from malignant SGTs (*p* = 0.006) (Table 4 and Figure 3).

**Figure 4:**
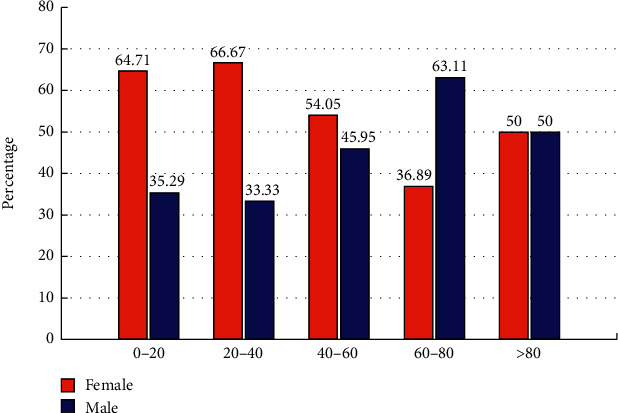
Gender distribution of benign and malignant SGTs in different age groups (20 year intervals).

As Figure 4 shows, the proportion of male and female patients was significantly different among age groups. While the percentage of females decreased from 0-20 to 60-80 age groups, the proportion of males increased, *p* < 0.001. We had 8 patients within the 80-100 group that were equally distributed between the two sex groups.

### 3.4. Tumor Size

The size of SGTs varied between benign and malignant tumors and across different sites. The mean tumor size was 3.28 cm ± 1.58, ranging from 0.5 to 10 cm. The mean size of major SGTs was 3.3 cm ± 1.57. Minor SGTs had smaller tumor sizes (2.8 cm ± 1.68), but the difference was not statistically significant (*p* = 0.310). Additionally, there was no clear difference in tumor size between benign and malignant tumors (3.25 cm ± 1.47 and 3.39 cm ± 1.85 for benign and malignant, respectively).

## 4. Discussion

SGTs are a rare group of tumors with diverse histological subtypes arising from the major salivary glands and minor salivary glands of the oral cavity and upper aerodigestive tract. Due to the rarity, histological heterogeneity, and location diversity, epidemiological studies of SGTs are challenging for researchers [28].

SGT epidemiological data are not well documented, owing to substantial variation in the incidence and prevalence of these tumors between ethnic groups, suggesting a geographical diversity in the frequency of SGTs [5, 6]. In addition, due to the absence of cancer registries in many parts of the world, the majority of epidemiological data on SGTs are based on single hospital or multiple institution databases [3, 24, 29–31] and are not true population-based investigations. This can also contribute to variation in the frequency of SGTs.

Various investigations have been undertaken in recent years to determine the epidemiology of SGTs. However, many of these studies were merely descriptive studies [3, 5, 21, 30, 32–34]. In this regard, in the current retrospective study, we looked at the epidemiology of SGTs in the southern population of Iran by making statistical comparisons wherever possible.

Several previous studies indicated that females are more likely than males to develop SGTs [20, 22, 30, 32, 35]. However, variations exist between tumor subtypes [24, 29, 31, 32]. In this study, the majority of cases (54.1%) were females, which was in accordance with the literature, including previous reports from Iran [3, 24, 31].

The majority of cases in the current research were benign (74.5%), consistent with the previous studies indicating that benign tumors comprise 55.5 to 88.6% of all SGTs [20, 22, 29, 35]. However, previous reports from African countries revealed a higher prevalence of malignant tumors, suggesting that the prevalence of SGTs may be influenced by geographical conditions [36, 37]. Similarly, previous studies from midwest, south, center, and northwest parts of Iran conducted between 2007 and 2013 also showed that benign tumors accounted for the majority of SGTs [3, 31, 34, 38]. However, the epidemiological investigation of minor SGTs by Taghavi et al. in 2016, which was conducted in Tehran, capital city of Iran (located in the north), showed 64.7% of patients diagnosed with malignant SGTs, the majority of which originated from minor salivary glands (73.9%) [24]. As indicated by previous reports, tumors of minor salivary glands were more likely to be malignant [22, 39–41]. Therefore, differences in malignancy rate can be explained.

In addition, the distribution of benign and malignant SGTs was similar between genders with 25.8% of males and 25.1% of females having malignant tumors, which was similar to the previous reports [20, 22, 31, 42]. However, variations exist concerning the gender distribution of benign and malignant SGTs in the literature [6, 29, 30, 43].

Among the heterogeneous histologic types of benign SGTs, pleomorphic adenoma exhibits the highest prevalence, constituting 52.5% of all cases and comprising a substantial 70.5% of all benign SGTs. Warthin's tumor was the second most common tumor with 16% of all SGTs and 21.5% of benign SGTs. These findings are almost consistent with the preexisting literature [3, 7, 22, 29–31, 35, 43]. Meanwhile, in a few studies, basal cell adenoma or myoepithelioma has been indicated as the second most prevalent benign SGT [6, 24, 38, 44].

The most common malignant SGTs were mucoepidermoid carcinoma and adenoid cystic carcinoma, accounting for 26.2% and 22.3% of malignant tumors, respectively. This was in accordance with previous reports from Iran [3, 23, 24, 31, 33] and other parts of the world [20, 22, 42]. Generally, mucoepidermoid carcinoma, adenoid cystic carcinoma, acinic cell carcinoma, and adenocarcinoma were the most frequent/common malignant SGTs reported in the literature [21, 29, 32, 45].

Regarding anatomic location, recent studies suggest that 51–85% of all SGTs originate from the parotid gland, 7.5–10% from the submandibular gland, 0–2.5% from the sublingual gland, and 7.5–27% from minor salivary glands [20, 22, 29]. In the present study, the majority of SGTs arose from major salivary glands (96.2%), with the parotid gland as the most common site (83.7%), followed by the submandibular gland (12.3%). Minor SGTs comprised 3.7% of all SGTs, with the majority of them originating from buccal mucosa (33.3%), followed by the floor of the mouth (20%) and palate (20%), which collectively support the previous reports. According to the literature, the palate is the most common site of involvement for minor SGTs. The second and third most common sites of involvement vary in the literature, including the buccal mucosa, retromolar area, and tongue. The current research was conducted within the ENT department, and it is noteworthy that minor SGTs are commonly managed by maxillofacial surgeons due to their specific site of involvement. This could result in referral bias and underrepresentation of these tumors in our study. Also, the number of cases (15 minor SGTs) might be a relatively small sample size compared to previous studies, which can lead to variations in the results and the possibility that the palate's position as the second most common site was influenced by chance [28, 30, 42, 46].

According to the literature, benign and malignant SGTs are more likely to develop in certain anatomical sites. For instance, the majority of SGTs occurreing in parotid gland (68.7-91%), and submandibular gland (64-89%) are benign tumors [7, 20, 22, 29, 47]. On the contrary, tumors of the sublingual gland were most commonly found as malignant (75%-100%) [7, 22, 28, 47, 48]. In the present investigation, a significant association was observed between the tumor's site and its malignancy status. The majority of SGTs arising from the major salivary glands were found to be benign, with parotid tumors and submandibular gland tumors comprising 78.4% and 72.0% of such cases, respectively. Conversely, all cases originating from the minor salivary glands were identified as malignant (*p* < 0.001). These findings are consistent with those of previous studies [5, 46].

The frequency of malignant minor SGTs varies in the literature, ranging from 26% to 61.9% [7, 20, 22, 28]. In the present investigation undertaken at the ENT department of two major hospitals in Shiraz, 15 cases of minor SGTs were identified, constituting 3.7% of all SGTs. Notably, all of these cases were malignant. These findings are in accordance with the previous ones conducted at various hospitals and clinical centers, which observed a higher propensity for malignancy among minor SGTs. Nevertheless, a significant disparity emerges when comparing these findings with studies carried out at pathology centers, where a lower proportion of malignant minor SGTs has been reported. This discrepancy may potentially be attributed to the involvement of distinct patient populations, wherein dental surgery outpatient clinics might encounter a higher number of benign minor SGTs due to routine oral health check-ups and dental procedures. Consequently, a greater representation of benign cases in these studies could lead to an underestimation of the true epidemiology of malignant minor SGTs in pathology-based investigations. In fact, establishment of cancer registries could significantly enhance the existing knowledge on SGTs' epidemiology, allowing for a more comprehensive analysis of their incidence and prevalence patterns [4, 5, 33, 49, 50].

Sublingual gland tumors are extremely uncommon and predominantly identified as malignant. Over the course of an 8 year study period, only one case of a malignant sublingual gland tumor was identified among all the cases examined [20, 29, 40].

In our study population, benign and malignant SGTs were observed among all age groups (7-95 years) with a mean age of 47.4 ± 16.2 years which was in accordance with previous studies [5, 22, 30]. While it appeared that most of SGTs occurred between 40 and 60 years, some variations existed; for instance, pleomorphic adenoma was most prevalent in individuals aged 20-40 years, and Warthin's tumor was most common in 60-80 years.

We observed a statistically significant association between a tumor's benign/malignant status and patients' ages at diagnosis in the current investigation. While the younger population was more prone to benign tumors, a higher percentage of older ones suffered from malignant SGTs (*p* = 0.006). Oliveira et al. also reported a similar trend in a retrospective study from Brazil. Their study included 599 SGT patients with a peak incidence between 30 and 39 years. The study indicated that malignant tumors had a significantly higher median age compared with benign ones [4]. In another study from Brazil, Fonseca et al. found that patients with malignant tumors were about ten years older than those with benign tumors [5]. However, Gontarz et al. reported an increased incidence of salivary malignant tumors in younger patients (<19 years old) compared with the adult group [51]. Similar findings were also reported by Louredo et al. in a systematic review, which demonstrated a higher frequency of malignant tumors (75.4% of all SGTs) in the studied population (2937 cases younger than 19 years old). These contradictory results could be due to the data collection from the studies carried out in oncology centers, leading to higher reports of malignant tumors. Another possibility could be that there is a tendency to report malignant tumors in the articles as a result of publication bias [52]. Some reports also indicated that there was no significant association between age and malignancy status of SGTs [3, 22]. Such differences can be explained by differences in methodological approaches, study population features, and the interval time that has been selected for the study.

In addition, the proportions of male and female patients were significantly different among age groups. While the percentage of females decreased from 0-20 to 60-80 year old groups, the proportion of males increased (*p* < 0.001). It may indicate that while men are more prone to develop SGTs at older ages of life, females are at higher risk of SGTs at younger ages.

Tumor size is also considered an important predictor of distant metastasis and overall prognosis [53, 54]; metastases occur in 7% of patients with tumors less than 4 cm but in 20% of instances with tumors greater than 4 cm [55]. Similarly, a smaller tumor size is associated with a more favorable prognosis [56–59].

In our study, the mean size of tumors was 3.28 cm ± 1.58 (0.5 to 10 cm). We found no significant differences between the size of SGTs of major and minor glands, as well as the size of benign and malignant tumors.

To the best of our knowledge, tumor size is hardly reported in the literature. Radomski et al. investigated the tumor size among 256 children diagnosed with malignant SGTs. The study showed a mean tumor size of 2.3 cm ± 1.2 [60]. In another study by da Silva et al. on 2292 cases of SGTs, tumor size ranged from 0.3 to 15 cm. The study observed a larger mean tumor size in malignant than benign tumors [6].

In summary, the findings of the current study were mainly consistent with the existing literature. There was a small female predilection in the frequency of SGTs. The majority of cases were benign tumors. Pleomorphic adenoma and mucoepidermoid carcinoma were the most common benign and malignant SGTs.

Our study observed a number of significant findings. The majority of cases which developed from the major salivary glands were benign, while all minor SGTs were malignant. Benign tumors most commonly developed from the parotid gland. Patients were more likely to develop malignant tumors in older age. In our study, the proportions of male and female patients were significantly different among different age groups.

## Figures and Tables

**Table 1:** Distribution of benign and malignant SGTs according to age, gender, tumor behavior, and tumor location.

Characteristics	*N* (%)	Mean
Age (years)		
Total	405 (100)	47.4
0-20	20 (4.9)	16.5
20-40	133 (32.8)	32.1
40-60	152 (37.5)	51.1
60-80	93 (22.9)	66.8
>80	7 (1.7)	83.7
Gender		
Female	219 (54.1)	
Male	186 (45.9)	
Benign/malignant		
Benign	302 (74.5)	
Malignant	103 (25.4)	
Tumor location		
Major salivary gland		
Parotid	339 (83.7)	
Submandibular	50 (12.3)	
Sublingual	1 (0.2)	
Total	390 (96.2)	
Minor salivary gland	15 (3.7)	

**Table 2:** Histopathology and gender distribution of benign and malignant SGTs.

	*N* = 405	%^a^	%^b^	Sex			
				Male		Female	
				*n*	%^a^	*n*	%^a^
Benign							
Pleomorphic adenoma	213	52.5	70.5	69	17	144	35.5
Warthin's tumor	65	16	21.5	59	14.5	6	1.4
Basal cell adenoma	15	3.7	4.9	5	1.2	10	2.4
Myoepithelioma	4	0.9	1.3	2	0.4	2	0.4
Oncocytoma (oxyphilic adenoma)	3	0.7	0.9	2	0.4	1	0.2
Monomorphic adenoma	2	0.4	0.6	1	0.2	1	0.2
Total	302	74.5	100	138	34	164	40.4
Malignant							
Mucoepidermoid carcinoma	27	6.6	26.2	11	2.7	16	3.9
Adenoid cystic carcinoma	23	5.6	22.3	7	1.7	16	3.9
Acinic cell carcinoma	14	3.4	13.5	6	1.4	8	1.9
Salivary duct carcinoma	8	1.9	7.7	6	1.4	2	0.4
Lymphoma	6	1.4	5.8	2	0.4	4	0.9
Malignant mixed tumor	5	1.2	4.8	2	0.4	3	0.7
Carcinoma ex pleomorphic adenoma	4	0.9	3.8	2	0.4	2	0.4
Basal cell adenocarcinoma	3	0.7	2.9	1	0.2	2	0.4
Poorly differentiated carcinoma	3	0.7	2.9	2	0.4	1	0.2
Epithelial myoepithelial carcinoma	3	0.7	2.9	2	0.4	1	0.2
Hyalinizing clear cell carcinoma	1	0.2	0.9	1	0.2	0	0
Oncocytic carcinoma	1	0.2	0.9	1	0.2	0	0
Papillary carcinoma	1	0.2	0.9	1	0.2	0	0
Papillary adenocarcinoma	1	0.2	0.9	1	0.2	0	0
Adenocarcinoma	1	0.2	0.9	1	0.2	0	0
Small cell neuroendocrine carcinoma	1	0.2	0.9	1	0.2	0	0
Adenosquamous cell carcinoma	1	0.2	0.9	1	0.2	0	0
Total	103	25.4	100	48	11.8	55	13.5

^a^Percentage in relation with total number of cases. ^b^Percentage in relation with the group (benign/malignant).

**Table 3:** Distribution of benign and malignant SGTs according to primary sites of involvement.

		Major salivary glands			Minor salivary glands					NS
		Parotid	Submandibular	Sublingual	Palate	Retromolar	Buccal	Floor of the mouth	Tongue	
Benign	Pleomorphic adenoma	178	35	0	0	0	0	0	0	0
	Warthin's tumor	65	0	0	0	0	0	0	0	0
	Basal cell adenoma	14	1	0	0	0	0	0	0	0
	Myoepithelioma	4	0	0	0	0	0	0	0	0
	Oncocytoma	3	0	0	0	0	0	0	0	0
	Monomorphic adenoma	2	0	0	0	0	0	0	0	0
	Total^a^	266	36	0	0	0	0	0	0	0
	%^a^	88	11.9	0	0	0	0	0	0	0

Malignant	Mucoepidermoid carcinoma	20	4	0	0	1	0	1	0	1
	Adenoid cystic carcinoma	10	5	1	3	0	1	2	1	0
	Acinic cell carcinoma	13	0	0	0	0	1	0	0	0
	Salivary duct carcinoma	8	0	0	0	0	0	0	0	0
	Lymphoma	3	2	0	0	0	1	0	0	0
	Malignant mixed tumor	4	0	0	0	0	1	0	0	0
	Carcinoma ex pleomorphic adenoma (CXPA)	3	1	0	0	0	0	0	0	0
	Basal cell adenocarcinoma	2	0	0	0	0	1	0	0	0
	Poorly differentiated carcinoma	3	0	0	0	0	0	0	0	0
	EMC	3	0	0	0	0	0	0	0	0
	Hyalinizing clear cell carcinoma	0	0	0	0	1	0	0	0	0
	Oncocytic carcinoma	1	0	0	0	0	0	0	0	0
	Papillary carcinoma	0	1	0	0	0	0	0	0	0
	Papillary adenocarcinoma	1	0	0	0	0	0	0	0	0
	Adenocarcinoma	0	1	0	0	0	0	0	0	0
	Small cell neuroendocrine carcinoma	1	0	0	0	0	0	0	0	0
	Adenosquamous cell carcinoma	1	0	0	0	0	0	0	0	0
	Total^a^	73	14	1	3	2	5	3	1	1
	%^a^	70.8	13.5	0.9	2.9	1.9	4.8	2.9	0.9	0.9

Total	*n*(%)^b^	339(83.7)	50(12.3)	1(0.2)	3(0.7)	2(0.4)	5(1.2)	3(0.7)	1(0.2)	1(0.2)

^a^Percentage in relation with the group (benign/malignant). ^b^Percentage in relation with total number of cases.

**Table 4:** Distribution of benign and malignant salivary gland tumors according to the primary site of involvement, gender, and age groups.

	Benign		Malignant		Total		*p* value
	*n*	%	*n*	%	*n*	%	
Anatomical site							
Parotid gland	266	78.4	73	21.5	339	83.7	.000
Submandibular gland	36	72	14	28	50	12.3	
Sublingual gland	0	0	1	100	1	0.2	
Minor salivary gland	0	0	15	100	15	3.7	
Gender							
Female	164	74.8	55	25.1	219	54.1	.481
Male	138	74.1	48	25.8	186	45.9	
Age (years)							
0-20	15	88.2	2	11.7	17	4.1	.006
20-40	106	82.1	23	17.8	129	31.8	
40-60	109	73.6	39	26.3	148	36.5	
60-80	69	66.9	34	33	103	25.4	
>80	3	37.5	5	62.5	8	1.9	

**Table 5:** Distribution of benign and malignant SGTs in different age groups (intervals of 20 years).

		Age range	Mean age	Age groups (years)					Total	
				0-20	20-40	40-60	60-80	>80	*n*	%^b^
				*n*(%)^a^						
Benign tumors	Pleomorphic adenoma	7-74	40.5	14(6.5%)	103(48.3%)	68(31.9%)	28(13.1%)	0(0%)	213	52.5
	Warthin's tumor	29-81	59.2	0(0%)	2(3%)	29(44.6%)	32(49.2%)	2(3%)	65	16
	Basal cell adenoma	33-70	54.1	0(0%)	1(6.6%)	8(53.3%)	6(40%)	0(0%)	15	3.7
	Myoepithelioma	16-56	40	1(25%)	0(0%)	3(75%)	0(0%)	0(0%)	4	0.9
	Oncocytoma	70-74	71.6	0(0%)	0(0%)	0(0%)	3(100%)	0(0%)	3	0.7
	Monomorphic adenoma	48-83	65.5	0(0%)	0(0%)	1(50%)	0(0%)	1(50%)	2	0.4

Malignant tumors	Mucoepidermoid carcinoma	22-95	52.6	0(0%)	6(22.2%)	11(40.7%)	7(25.9%)	3(11.1%)	27	6.6
	Adenoid cystic carcinoma	25-72	49.8	0(0%)	7(30.4%)	8(34.7%)	8(34.7%)	0(0%)	23	5.6
	Acinar cell carcinoma	18-67	39.1	1(7.1%)	6(42.8%)	6(42.8%)	1(7.1%)	0(0%)	14	3.4
	Salivary duct carcinoma	45-78	62.2	0(0%)	0(0%)	2(25%)	6(75%)	0(0%)	8	1.9
	Lymphoma	11-74	58.3	1(16.6%)	0(0%)	1(16.6%)	4(66.6%)	0(0%)	6	1.5
	Malignant mixed tumor	50-81	63.4	0(0%)	0(0%)	2(40%)	2(40%)	1(20%)	5	1.2
	Carcinoma ex pleomorphic adenoma	31-67	52.5	0(0%)	1(25%)	2(50%)	1(25%)	0(0%)	4	0.9
	Basal cell adenocarcinoma	53-62	57.6	0(0%)	0(0%)	2(66.6%)	1(33.3%)	0(0%)	3	0.7
	Poorly differentiated carcinoma	27-76	52	0(0%)	1(33.3%)	1(33.3%)	1(33.3%)	0(0%)	3	0.7
	Epithelial myoepithelial carcinoma	36-82	60.6	0(0%)	1(33.3%)	0(0%)	1(33.3%)	1(33.3%)	3	0.7
	Hyalinizing clear cell carcinoma	36-36	36	0(0%)	1(100%)	0(0%)	0(0%)	0(0%)	1	0.2
	Oncocytic carcinoma	67-67	67	0(0%)	0(0%)	0(0%)	1(100%)	0(0%)	1	0.2
	Papillary carcinoma	58-58	58	0(0%)	0(0%)	1(100%)	0(0%)	0(0%)	1	0.2
	Papillary adenocarcinoma	58-59	59	0(0%)	0(0%)	1(100%)	0(0%)	0(0%)	1	0.2
	Adenocarcinoma	47-47	47	0(0%)	0(0%)	1(100%)	0(0%)	0(0%)	1	0.2
	Small cell neuroendocrine carcinoma	77-77	77	0(0%)	0(0%)	0(0%)	1(100%)	0(0%)	1	0.2
	Adenosquamous cell carcinoma	43-43	43	0(0%)	0(0%)	1(100%)	0(0%)	0(0%)	1	0.2

^a^Percentage in relation with the histopathologic group. ^b^Percentage in relation with total number of cases.

## Data Availability

The data that support the findings of this study are available on request from the corresponding author.
